# Multimodal Microscopy of Partially Oriented *para*-Hexaphenylene Nanoaggregates

**DOI:** 10.1021/acs.langmuir.4c03073

**Published:** 2024-11-23

**Authors:** Frank Balzer, Mario Fratschko, Roland Resel, Manuela Schiek

**Affiliations:** † SDU Centre for Photonic Engineering, University of Southern Denmark, Alsion 2, 6400 So̷nderborg, Denmark; ‡ Institute of Solid State Physics, Graz University of Technology, Petersgasse 16, 8010 Graz, Austria; § Center for Surface- and Nanoanalytics (ZONA), Johannes Kepler University Linz, Altenberger Str. 69, 4040 Linz, Austria; ∥ Institute for Physical Chemistry (IPC) & Linz Institute for Organic Solar Cells (LIOS), Johannes Kepler University, Altenberger Str. 69, 4040 Linz, Austria

## Abstract

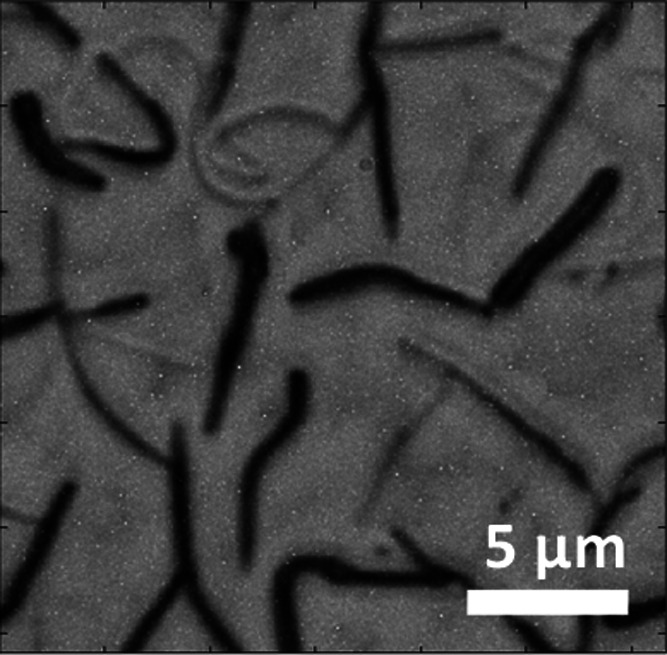

Organic molecular beam deposition (OMBD) of *para*-hexaphenylene (p6P) on polycrystalline platinum results in the formation of unique nanoaggregates, predominantly as nanofibers and nanoribbons. These aggregates exhibit distinct morphological properties, as revealed by atomic force microscopy (AFM). Grazing incidence X-ray diffraction (GIXD) confirms the p6P herringbone structure as partially oriented aggregates with a bias of previously observed contact planes parallel to the substrate. The optical properties of the aggregates are analyzed using polarization microscopy, fluorescence microscopy, and Raman microscopy to distinguish three different aggregate types with a focus on aspects such as molecule orientation within the aggregates, including those lying and standing upright. Polarized microscopy indicates that the molecular orientation within the fluorescing, fiber-like aggregates is generally perpendicular to the long fiber axis and parallel to the substrate, which seems not to be the case for the other two types. This finding is crucial for applications utilizing p6P’s polarized emission, such as in photonic and optoelectronic devices.

## Introduction

**Figure 1:**
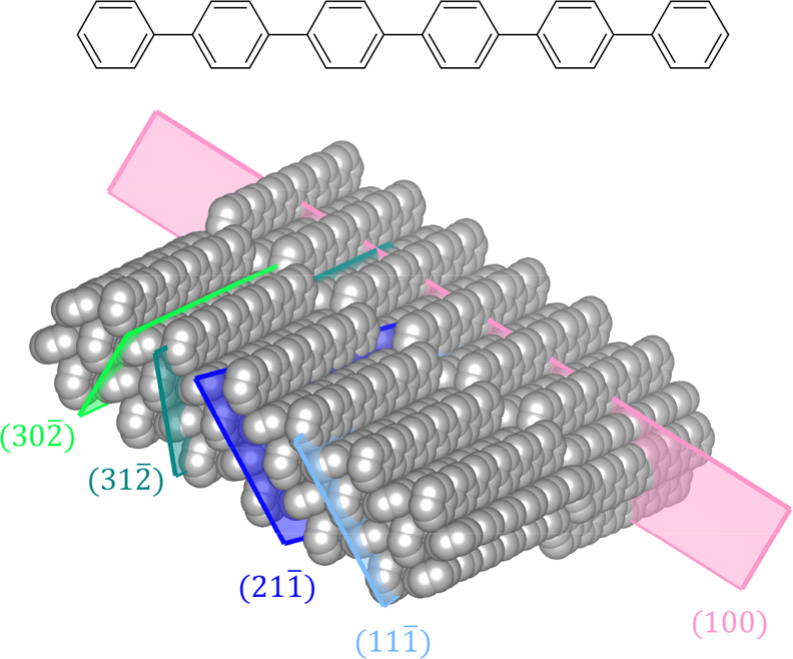
Structural formula and crystal structure of *para*-hexaphenylene (p6P), together with prominent planes observed by GIXD.

Small conjugated organic molecules such as the *para*-phenylenes,^1−4^ where the prototypical *para*-hexaphenylene (p6P) is depicted in Figure 1,^5^ phenylene/thiophenes,^6^ or others^7−9^ are a unique class of nanomaterials that have gained significant attention over the last three decades due to their exceptional properties and wide-ranging applications.^10^ A significant characteristic of these materials is their ability to be customized via chemical modification, meaning that their properties can be altered by adding or substituting particular functional groups.^11,12^ In photonics, organic nanofibers have shown promise in the fabrication of waveguides, lasers, and other photonic devices.^13−16^ Their waveguiding properties have been used to create self-assembled microring cavities^17^ and other compact photonic units such as heterostructure nanowires and branched crystallites for logic gates,^18−21^ which might be essential components in future optical communication systems and quantum information devices. The performance of these devices often depends on the orientation of the molecules within the aggregates relative to the substrate and device geometry. Understanding and controlling molecular orientation is therefore crucial for optimizing their performance.^22^

Some optical nanofibers show strong faceting, either in-plane such as a methoxy-functionalized quaterphenylene (MOP4) on muscovite mica,^12^ or out-of-plane such as p6P on Au.^23^ Nanofibers at surfaces can have different internal morphologies, depending on the crystal growth at surfaces. Single crystalline aggregates have been observed on weakly interacting surfaces^24,25^ as well as aggregates with polycrystalline character.^26^ However, the relationship between morphology, molecular orientation within, and their optical properties remains not fully understood, particularly on polycrystalline substrates. Grown on Ag, p6P nanofibers have been shown to exhibit polarization-insensitive plasmonic activity, which is beneficial for certain optoelectronic applications.^27^ While Ag was used due to its effectiveness in plasmonic applications involving the blue fluorescence from p6P nanofibers, Pt is also a suitable substrate, for instance, due to its inertness. It tends to support a more diverse variety of aggregates,^27^ but it remained unclear whether these represent distinct polymorphs, a single polymorph with different substrate contact faces, or a combination of both.

The orientation of single molecules and unit cell axes within nanofibers has been optically investigated by, for example, polarized fluorescence microscopy and their linear birefringence, respectively.^28^ Raman spectroscopy is another technique that has been widely used to probe the properties and orientation of molecules in organic semiconductors,^29−33^ but often concentrating on the molecule level since the energy of lattice phonon modes is small, typically below 200 cm^–1^.^34−38^

Here, a multimodal approach will be used to bridge this knowledge gap, characterizing p6P nanoaggregates grown on polycrystalline Pt. The morphology and spatially resolved optical properties using AFM, GIXD, fluorescence, birefringence, optical reflectivity, and Raman activity are investigated with a particular focus on understanding the relationship between molecular orientation, morphologies, and optical behavior. By elucidating these relationships, we aim to provide insights for the development of p6P-based photonic and optoelectronic devices.

## Results and Discussion

**Figure 2:**
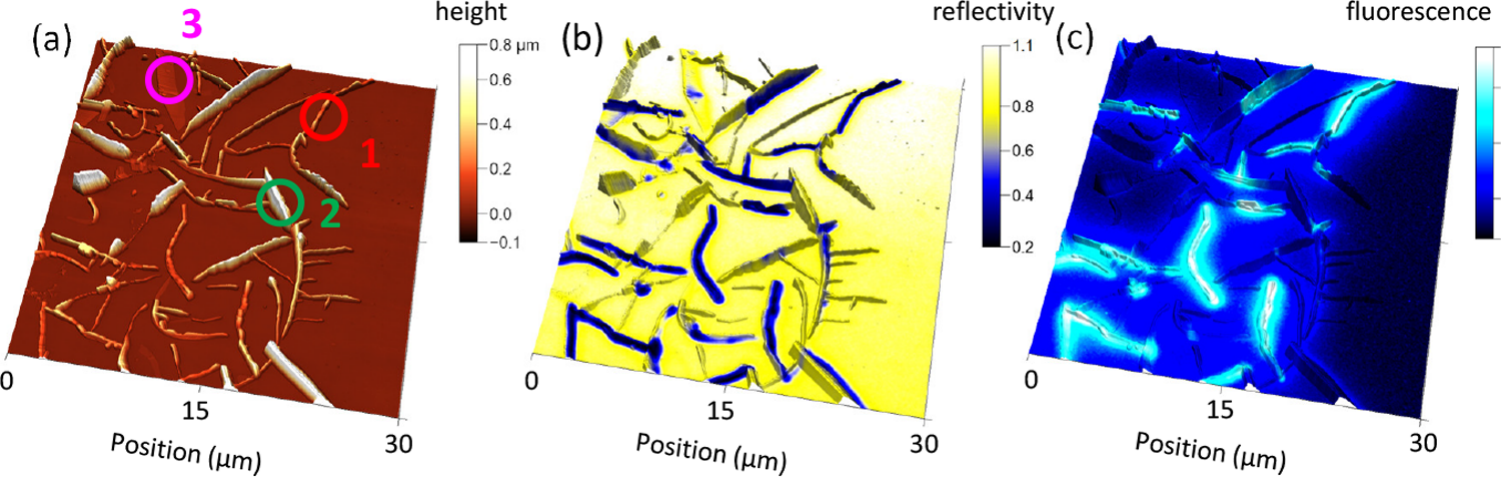
Multimodal microscopy of p6P aggregates. (a) AFM-image of nominally 20 nm p6P on Pt, deposited at 200 °C. The corresponding optical reflection microscope image and the fluorescence microscope image, both false color, are shown as overlays on the AFM image in (b) and (c), respectively. Different aggregate types are marked by numbers **1**, **2**, and **3**. The individual images together with a reflection image between crossed polarizers are shown in Figure S2 in the Supporting Information.

Organic molecular beam deposition (OMBD) of p6P at elevated temperatures onto polycrystalline Pt films leads to the formation of straight and bent fibers, along with ribbons, as previously observed through helium ion microscopy (HIM).^27,39^ A close examination using AFM reveals the variety of aggregates formed, illustrated in Figure 2a for a nominally 20 nm thick film deposited at a substrate temperature of 200 °C. These growth parameters were chosen because they guarantee the presence of aggregates with a number density and size distribution suitable for the available optical and morphological characterization techniques; see below. The length of the aggregates significantly exceeds the typical size of the underlying Pt grains, Figures S1a,b. Three distinct types of aggregates can be identified by their overall morphology: intermediate fibers (**1**, up to a few hundred nanometers in height) and tall fibers (**2**, several hundred nanometers up to a few micrometers in height), and wider flat ribbon-like entities (**3**). Type **2** aggregates often have a prismatic shape in AFM micrographs, where obtaining a stable image is more difficult compared with the other aggregate types. The type **2** aggregates from this work most likely resemble the previously identified tall ribbons standing on their small side.^27^ Some of these ribbons have even shown severe twisting along their long axis. The prism-like appearance in the AFM images is probably due to the convolution with the shape of the AFM tip, which has a half-cone angle of at least 20°.

**Figure 3:**
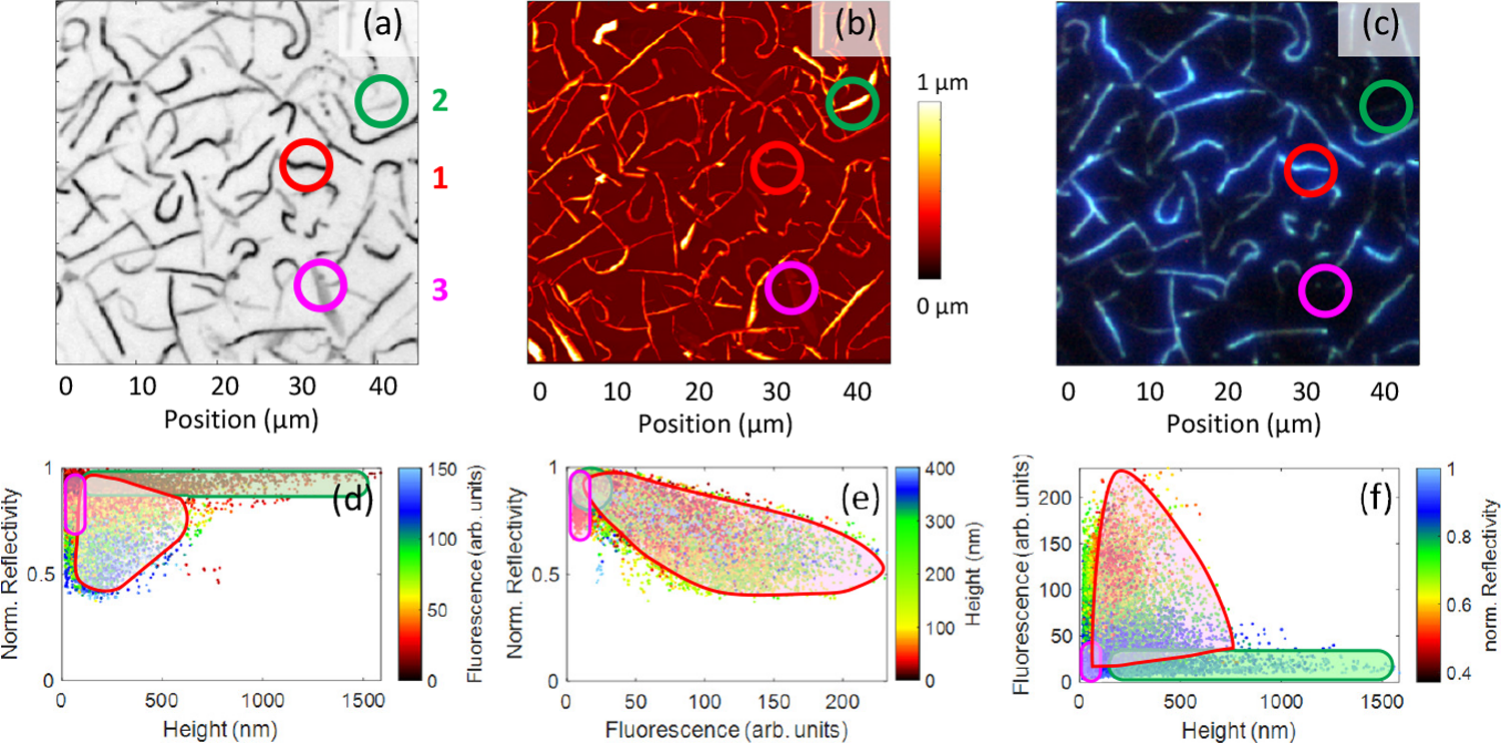
Correlations between gray scale reflection microscopy (a), AFM (b), and fluorescence microscopy (c). The color of the dots in (d–f) encodes the third parameter. The correlation between reflectivity and aggregate height (d) shows a minimum for aggregates around 200 nm tall. The larger the fluorescence intensity, the lower the reflectivity (e). Only the type **1** fibers show significant fluorescence, (f). Three representative aggregate types are indicated by colored circles in the microscopy images (red: intermediate height fiber, **1**; green: tall fiber, **2**; pink: flat ribbon, **3**. The results for the correlation of the different entities are indicated by areas of the same color. For types **1** and **2**, only pixels with at least 30% of the maximum aggregate height were considered to minimize edge effects; therefore, some points do not belong to either of the three areas.

Optical microscope images such as Figures 2b,c, S2b–d, and 3 further distinguish these morphologies based on their optical properties, such as reflectivity, fluorescence, and birefringence, in Table 1. More information about the dependence of the morphology on process parameters such as nominal film thickness and substrate temperature can be found in the Supporting Information, Figures S3–S6. The intermediate-height fibers show low reflectivity, strong birefringence (Figure S2d), and strong blue fluorescence. The taller type **2** structures have high reflectivity and strong birefringence but minimal fluorescence. Finally, the flat type **3** ribbons have high reflectivity, little fluorescence, and little birefringence. A correlation between reflectivity and aggregate height, Figure 3d, obtained from the corresponding images, Figure 3a,b reveals that fibers with heights between 50 and 400 nm have the lowest reflectivity.

**Table 1:** Morphological and Optical Properties of the Various Types of p6P Aggregates and the Descriptions ”Strong” and ”Weak” Relate to the Maximum Observed Values^a^

type	reflectivity	height (nm)	fluorescence	birefringence	Raman A–E bands
**1**	low	a few 100	strong	strong	strong
**2**	high	several 100 to a few 1000	very weak	strong	weak
**3**	high	several 10	very weak	very weak	n.a.

a Note that for the type **3** aggregates, Raman intensities have not been studied

Some of the aggregates exhibit characteristic fluorescence when excited with UV light, Figures S2c and 3c, with the emission spectrum predominantly situated in the UV to blue wavelength region.^40^ Notably, only medium-height needles (type **1**), appearing dark in the reflection image, show the typical fluorescence observed in other p6P/substrate combinations. The aggregate types **2** and **3** show negligible fluorescence. The less these fibers reflect light, the more they fluoresce, Figure 3e. The maximum fluorescence intensity comes from type 1 aggregates with about 100–400 nm height, Figure 3f. Finally, in between crossed polarizers, Figure S2d, the birefringence of the fibers (type **1** and **2**) manifests in bright conditions, whereas the ribbons are almost dark and thus mostly nonbirefringent. Note that for the extinction, the brightness of aggregates also depends on their orientation relative to the two polarizer orientations.

**Figure 4:**
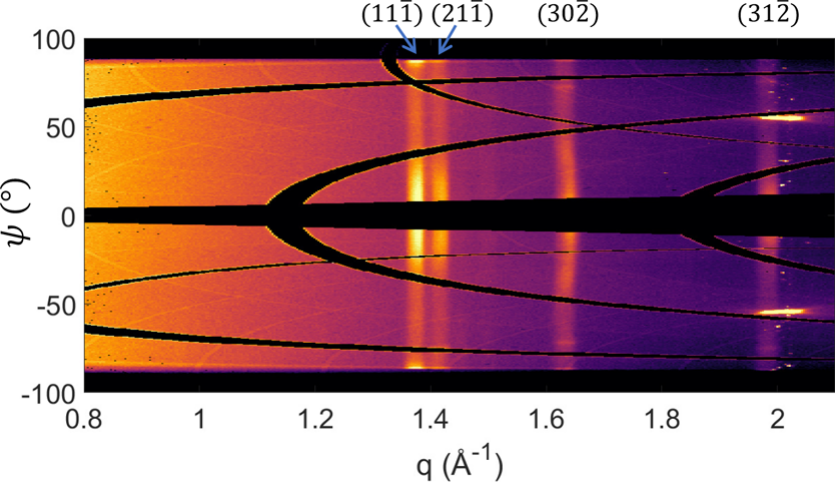
Reciprocal space map of a 40 nm thick p6P film on Pt (GIXD, incident angle 0.8°) plotted in cylindrical coordinates. Characteristic diffraction peaks arising from the net planes (1 1 1̅) (lattice spacing *d* = 4.59 *Å*, scattering vector *q* = 2π/*d* = 1.37 Å^–1^, (2 1 1̅) (*d* = 4.46 *Å*, *q* = 1.41 Å^–1^), (3 0 2̅) (*d* = 3.88 *Å*, *q* = 1.62 Å^–1^), and (3 1 2̅) (*d* = 3.16 *Å*, *q* = 1.99 Å^–1^) are observed. The strong diffraction peaks at *q* = 2.0 Å^–1^ and ψ = ± 54° arise due to 111 of the used silicon substrate. The reciprocal space plot in Cartesian coordinates is presented in the Supporting Information, Figure S7. Note the slight shift to higher *q* values for the (3 0 2̅) ring at ψ = 0°.

The crystallographic properties of the films are characterized by GIXD. In contrast to microscopy investigations, integral information across the sample is provided. The result is depicted in Figure S7 for a 20 nm and a 40 nm thick sample. The presence of the strongest diffraction peak of p6P^41^ together with Debye–Scherrer rings indicates that the p6P crystals are randomly distributed.^42,43^ However, in Figure 4, the diffractogram for the 40 nm film is plotted in cylindrical coordinates. That way, Debye–Scherrer rings appear along vertical lines at constant values of scattering vector *q*. An increased intensity in the specular direction at ψ = 0° is observed. This suggests that the crystallites are not entirely randomly oriented but show a bias toward the alignment of these planes with the substrate surface. Furthermore, for the (3 0 2̅) ring a shift to higher *q* values by 0.007 Å^–1^ (i.e., to smaller interplanar distances) is observed. Possible explanations could be a small variation of the herringbone packing due to mechanical stresses parallel to the substrate surface. It is tempting to identify the distorted crystallites with the preferred (3 0 2̅) contact plane with the twisted type **2** aggregates.

The observation that only type **1** fibers fluoresce suggests that these aggregates alone have a significant component of the transition dipole for blue fluorescence parallel to the substrate. Note that for rodlike *para*-phenylene molecules with herringbone packing, the angle of maximum fluorescence intensity typically aligns with the net direction of the molecular transition dipole, which for p6P is oriented along the molecule’s long axis.^40,44^ In contrast, ribbons likely consist of molecules oriented with their long molecule axes perpendicular to the substrate, which is another typical growth mode of p6P.^1,45−47^ Because here the transition dipole is perpendicular to the substrate, they do appear dark under normal incidence UV illumination. For type **2** aggregates, the lack of fluorescence is not that straightforward to explain, because either they have to consist of upright standing molecules or the fluorescence is quenched because of, e.g., the tilting or stress.

**Figure 5:**
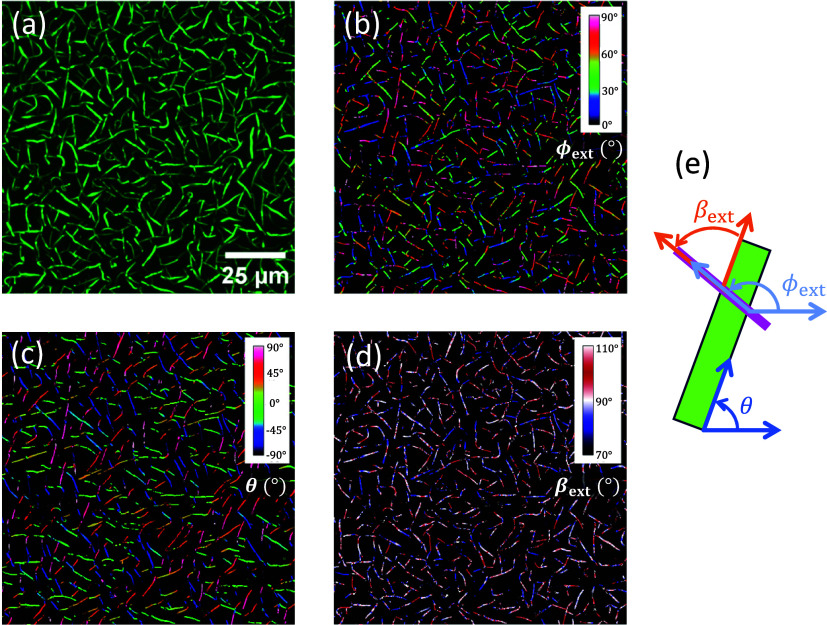
Polarization analysis of p6P fibers on poly crystalline Pt. A series of microscope images in reflection and between crossed polarizers using λ = 550 nm light is analyzed with respect to the local extinction angles ϕ_ext_ (b). The averaged optical microscope image (a) shows only the birefringent fibers. Together with the local orientation angle θ of the fibers’ long axes (c), the local extinction angles β_ext_ are obtained (d). All angles are defined in (e). The green rectangle symbolizes the aggregate, and the pink line a polarizer direction where the extinction is zero.

Thus, only fibers of type **1** could resemble the fibers previously observed on muscovite mica. This assumption is further supported by a polarization analysis of the fibers using linearly polarized light of λ = 550 nm, as shown in Figure 5. This wavelength has been chosen because it is far away from the absorption band of p6P in the UV and close to the wavelength of λ_exc_ = 532 nm used for the Raman scattering experiments; see below. The reflection microscopy image from Figure 5a is an average of 72 different images obtained by rotating the sample in between crossed linear polarizers over 360°, thus showing only the birefringent fibers, no Pt substrate or type **3** ribbons. Illuminating and detecting the reflected light through crossed polarizers for a rotational series of images deliver for each image pixel the local extinction angle ϕ_ext_, i.e., the polarizer angle where the reflected light is minimum, Figure 5b. The local fiber orientation θ, Figure 5c, is extracted following Rezakhaniha et al.^48^ These two pieces of information finally lead to the local extinction angle with respect to the long aggregate axis, β_ext_ = θ – ϕ_ext_, which is visualized in Figure 5d. For some of the aggregates, the extinction angle β_ext_ is constant; others seem to be assembled from subfiber-like components, i.e., they show domains of different extinction angles. All angles are defined in Figure 5e.

The local extinction angles of the fibers, β_ext_, average 90° with a fwhm of 20°. This means that for all identified aggregates the long unit cell axis is expected to be oriented approximately perpendicular (or parallel due to the 4-fold rotational symmetry) to the long aggregate axis. However, unlike polarized fluorescence, the extinction angle β_ext_ does not necessarily correspond to the long molecule axis orientation.^28^ The difference between the angle for the projected long molecule axis and the long unit cell axis can reach about 15° for the (2 1 1̅) contact plane.^3^ For extinction, the polarization angle is a property related to the symmetry of the crystal unit cell due to molecular excitonic interactions.^28,49^ To ultimately correlate optical extinction angle and molecular orientation, knowledge of the full dielectric tensor of p6P aggregates adopting the monoclinic crystal structure would be required.^50−52^

In summary, three distinct types of p6P aggregates were identified: intermediate-height fibers with strong blue fluorescence, tall fibers with high reflectivity but minimal fluorescence, and flat ribbons with little fluorescence or birefringence. A strong correlation between aggregate height and optical properties suggests that molecular orientation within these aggregates plays a key role, with the transition dipole for fluorescence likely aligned parallel to the substrate for type **1** fibers, and perpendicular for type **3** ribbons.

**Figure 6:**
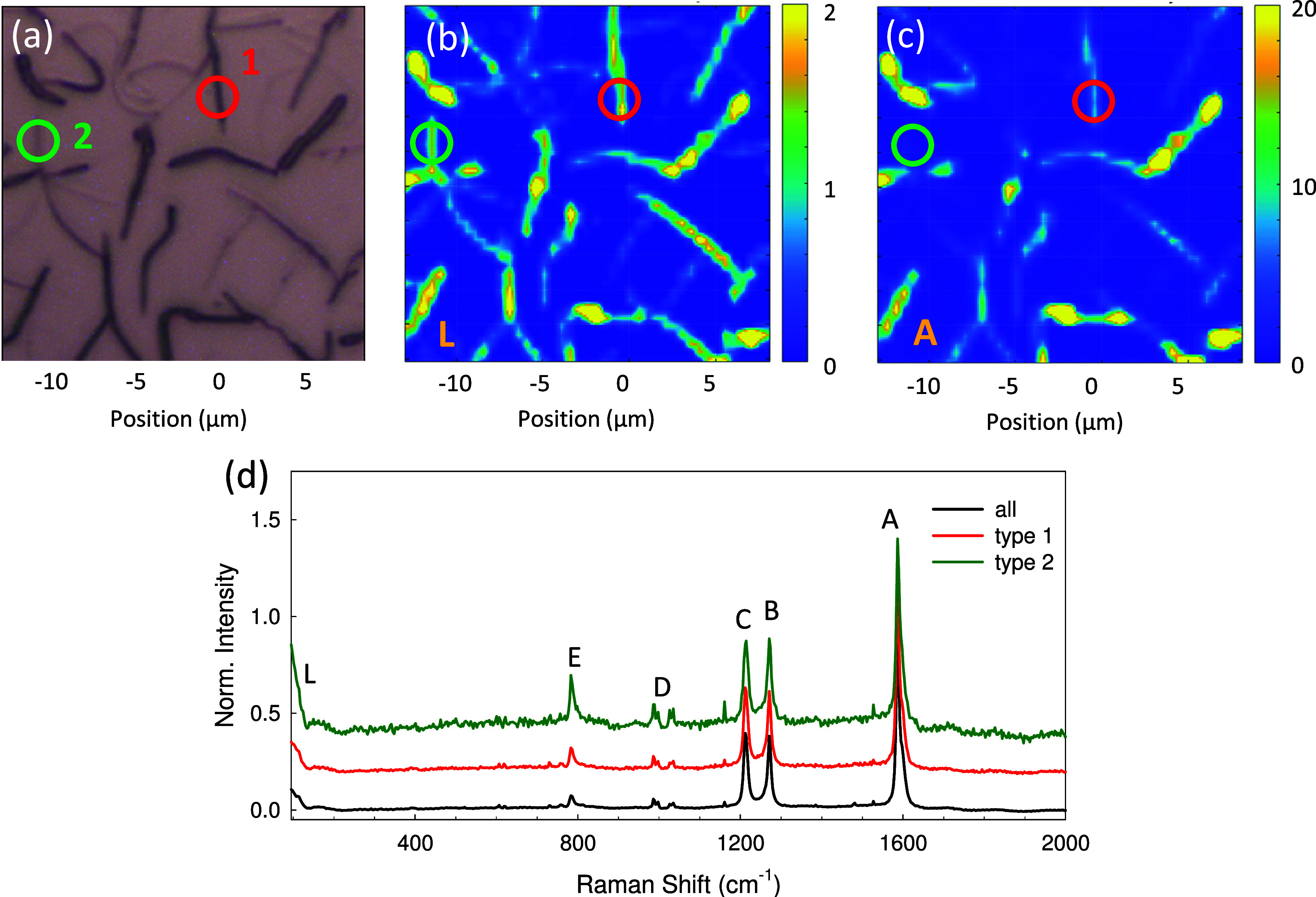
(a) Reflection microscopy and (b, c) corresponding confocal Raman images (green laser) of p6P fibers on Pt. Raman maps from the same area, excited with horizontally and vertically linear polarized light, have been summed up for that and integrated either over the librational band L (b), or over band A (c). In (d), spatially averaged Raman spectra are presented for the entire image (a) (black line), and for the two marked circular areas, i.e., for type **1** and type **2** aggregates. Prominent Raman bands are labeled with letters A–E and L. The Raman intensities in (d) have been normalized to the intensity of the A-band.

**Figure 7:**
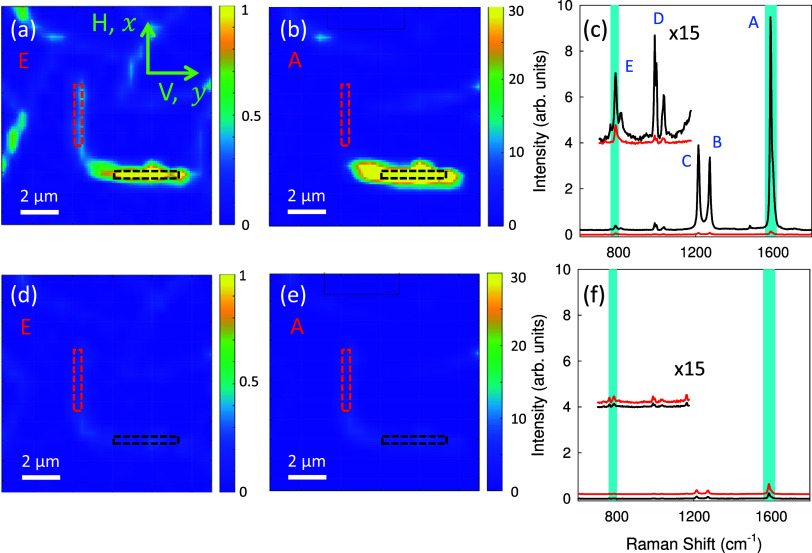
Raman polarization combination images of p6P fibers. The reflection microscope image in Figure 8 gives an overview over the mapped area: the L-shaped fiber is of type **1**. The light is H-polarized for the upper row and V-polarized for the lower row. In (a) and (d), the intensity of the E-band is shown; in (b) and (e), the intensity of the A-band is shown. Raman spectra for the two selected areas (dashed red and black rectangles) of perpendicularly oriented fibers are displayed in parts (c) and (f). The spectra of the two regions are slightly shifted along the *y*-axis, and the wavenumber regions between 700 and 1175 cm^–1^ are magnified by a factor of 15. The cyan areas mark the wavenumber regions plotted in the Raman microscope images. The bands are labeled according to Figure 6.

Confocal Raman microscope images obtained with green laser illumination of λ_exc_ = 532 nm together with corresponding optical images are shown in Figures 6, 7, S8, and S9. The most prominent bands are marked by the letters A–E and L. The Raman spectra, Figure 6d, agree within 10 cm^–1^ with spectra already published in the literature.^34,37,53^ Following Zhang et al., the bands A, B, and C are assigned to the ring C–C stretch vibration, the inter-ring C–C stretching, and the C–H in-plane bending, respectively. Band D is assigned to various origins including ring breathing, and band E to a ring deformation.^36,37,53,54^ Below 200 cm^–1^, librational ring motion determines the spectrum, band L,^54^ which can only partly be measured with the existing Raman setup. All bands A–E are expected to have A_g_ symmetry and, therefore, should be polarized. Note that the high-energy shoulder of band A has its origin in a Fermi resonance and that this shoulder has B_3g_ symmetry.^34,54^ Comparing the Raman map with the reflection microscope image, Figure 6a, suggests, that the dark fibers, i.e., the ones that absorb or scatter lots of light and also show strong fluorescence, lead to a strong Raman scattering signal, too, last column in Table 1. The intensity of the librational band L responds to both types of aggregates, but band A (along with the other two prominent bands B and C, shown in parts S8 and S9) is primarily sensitive to type **1** aggregates. This is also observed in the relative intensities of the various bands in the Raman spectra (Figure 6d).

**Figure 8:**
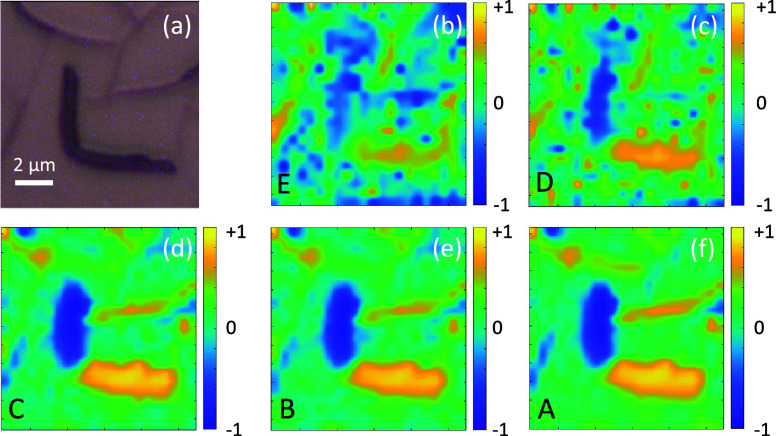
(b–f) Polarization ratios *P*_HV_ of the prominent bands from Figure 7. The corresponding optical reflection microscopy image is shown in (a).

As shown in Figure 7, various combinations of Raman polarization and detection are elucidated. The incident light is linearly polarized along the *x*-axis (H-polarized), and for the scattered light, either H- or V-polarization has been chosen. The corresponding reflection microscopy image, Figure 8a, provides an overview of the imaged sample. Two selected spectral ranges are depicted in Figure 7 – the intensity of the E band (a) and (d), and the intensity of the A-band, (b) and (e). In Figures 7c,f, Raman spectra are compared and averaged over the two selected aggregate areas for the H and V polarization combinations, respectively. As shown in Figure 6, the intensities of the D- and E-bands are less sensitive to the type of aggregate. For type **1**, the Raman bands have the highest intensity, when the polarization of the excitation is perpendicular to the long fiber axis shown in Figure 7c,f.

Similar Raman spectra have already been described for *p*-quaterphenylene,^55−57^ for p6P thin films^37,53^ and for p6P molecules.^36,54^ Polarized Raman spectroscopy has been used to determine molecule orientations in highly anisotropic organic crystallites.^37^ In particular, all of the Raman-active bands A–E have been identified to have A_g_ symmetry and therefore are polarized. With this, the measured polarization angle of the vibronic Raman modes serves as an indicator for the orientation of the long molecule axis within the aggregates. Based on the evaluation of their crystallographic orientation, the polarization of Raman modes for the p6P fibers is expected to be approximately perpendicular to their long axis. In fact, this is observed in the microscope images in Figure 7a,b.

A quantity to visualize the polarization dependence on aggregate type and orientation is the polarization ratio of the individual Raman peaks for H- and V-polarized excitation, *I*_H_ and *I*_V_, respectively^58^
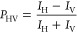
The polarization ratio can vary between −1 and +1, where for instance a value of +1 would imply a perfect polarization sensitivity of the Raman excitation along the H-direction, a value of −1 the same along the V-direction. As shown in Figure 8, these ratios are sketched for all bands from the sample shown in Figure 6. They reach ±0.95 for the type **1** aggregates, and their magnitude is maximal for horizontally and vertically aligned fibers, confirming the orientation of the long molecule axis being approximately perpendicular to the long fiber axis. The librational band on the other hand is polarized perpendicular to the long molecule axis. The ratios between the different bands for the sum of H- and V-polarization are significantly different for types **1** and **2** aggregates, Figure S10, also demonstrates their different optical properties. However, in order to better understand these effects, a more detailed polarization analysis is needed in the future.^59−61^

## Conclusions

Organic molecular beam deposition of p6P on polycrystalline Pt films leads to the formation of distinct nanoaggregates, primarily nanofibers and ribbons. Detailed analyses using AFM reveal that these aggregates exhibit varied morphologies, which are closely linked to their unique optical properties. Furthermore, GIXD confirms the presence of the well-known p6P herringbone structure. The observation of Debye–Scherrer rings indicates randomly oriented p6P crystallites across the sample, but with a bias of the (1 1 1̅), (2 1 1̅), (3 0 2̅), and (3 1 2̅) planes parallel to the substrate. The optical measurements reveal that the aggregates are not necessarily single crystalline. Type **1** aggregates fluoresce, i.e., are supposed to consist of lying molecules, whereas type **3** aggregates are probably from upright standing molecules. Type **2** aggregates hardly fluoresce. They can be thought of as ribbons standing on their small side and twisted, leading to various tilt angles and stress. Importantly, only medium-height type **1** fibers exhibit characteristics similar to those previously observed on muscovite and phlogopite mica. Type **3** aggregates likely consist of upright-standing molecules, explaining their lack of fluorescence. The exact structure of type **2** aggregates remains unclear due to their minimal fluorescence but strong light extinction, differing Raman band ratios compared to those of type **1** aggregates and their fiber-like morphology. The fact that the aggregates hardly fluoresce indicates that the fluorescence is strongly quenched.^4,62^

These insights into the random alignment and molecular arrangement within the aggregates provide clues for their use in optoelectronic applications. By controlling the deposition conditions, tailored nanoscale structures with optimized properties can be developed, especially for advanced photonic devices. The clear correlation between molecular orientation and optical properties highlights the potential of these materials in future technologies, especially in the development of advanced photonic devices. Further exploration of their varying capabilities, such as launching surface plasmon polaritons, is warranted.

## Materials and Methods

*Para*-hexaphenylene powder was purchased from TCI America. Thin films have been prepared by OMBD in high vacuum (base pressure 1 × 10^–7^ mbar) with a deposition rate of 0.02 nm/s, ranging from 5 to 40 nm nominal thickness. The nominal thickness and the deposition rate have both been monitored by a quartz microbalance (Inficon XTC/2). As substrates, thin polycrystalline Pt films on a Si-wafer have been chosen, and resistively heated from the back to 200 °C during p6P deposition. The Pt films were prepared by e-beam deposition; their morphology consisting of small grains is shown in an AFM image in Figure S1a. Their thickness has been confirmed by X-ray reflectivity (XRR) evaluating Kiessig fringes^63^ to be approximately 80 nm.

AFM images have been taken by a JPK NanoWizard in intermittent contact mode (BudgetSensors Tap300-G, Nanosensors NCH, and Nanosensors SSS-NCH cantilevers, all with a resonance frequency of about 300 kHz and a force constant of 42 N/m, but varying tip radii of 10, 8, and 2 nm, respectively). For image analysis, software from the AFM manufacturer and Gwyddion^64^ has been used.

The optical microstructure of the samples was characterized using a Leitz DMRME polarization microscope and a Nikon Eclipse TE 300 inverted microscope equipped either with a halogen lamp or a high-pressure Hg lamp. With the polarization microscope, a simple microscopic polarization analysis was obtained by rotating the sample via a computer-controlled rotation stage (Thorlabs PRM1Z8) in steps of 5° over 360°. The sample was illuminated with linearly polarized light of λ = 550 nm (Thorlabs bandpass filter, fwhm 10 nm), and the reflected light was sent through a crossed linear polarizer before it entered the microscope camera (PixeLINK PL-B873-CU). After realigning all images in ImageJ,^65,66^ the polarization direction was determined pixelwise, for which the reflected light is minimum (extinction angle) via a discrete Fourier transform.^67^ The orientation θ of the single fibers was, at the same time, determined by calculating the structure tensor.^48,68^ From this, the local extinction angle with respect to the long fiber axis, β_ext_, was determined,^28^ at 0° and 180°, meaning that the polarization direction is parallel to the long fiber axis.

The blue fluorescence of the aggregates was excited with the 365 nm Hg line using a Nikon LU Plan ELWD 50 × objective (NA 0.55) and was observed through the same objective with a PixeLINK PL-B873-CU camera mounted at the side port of the microscope.

Images taken from the same sample spot using different techniques were later aligned in Matlab using Image Processing Toolbox,^69^ or ImageJ.^66^ Confocal Raman microscope images were taken with a LabRAM Aramis VIS system, and Raman maps were spatially interpolated to fit the image sizes of the optical and AFM images. A green laser with up to 5 mW of 532 nm excitation light and a 100× objective were used. No bleaching or degradation of the fibers has been observed since the fibers mainly absorb light in the blue to UV range.^1^ The linear polarization of the excitation laser and the detection have been controlled by waveplates and linear polarizers. The discussed polarization combinations of incident and probed reflected light are parallel and cross-polarized, i.e., H- and V-polarized.

Grazing incidence X-ray diffraction (GIXD) measurements were performed at the beamline XRD1, synchrotron Elettra, Trieste.^43,70^ The wavelength of the primary X-rays was 1.40 Å, and the diffracted beam was detected with a Pilatus 2 M detector located at a distance of 200 mm from the sample. For the measurement, an incidence angle of 0.8° was selected. The sample was rotated during measurement for 360° with a 60 s illumination time to enhance statistical accuracy. Data processing was performed using the software GIDVis^71^ representing the data as a function of the wave vector using the scattering vector . The experimental data are plotted in polar coordinates as a function of *q* versus the angle ψ in the range −90° to +90°; ψ = 0° corresponds to the specular diffraction geometry with the scattering vector perpendicular to the substrate surface. Enhanced intensities of the diffraction peaks at ψ = ± 90° arise from a dynamic scattering effect (Yoneda peak). The black areas in the reciprocal space maps are caused by the blind spots of the detector and the missing wedge around the origin of *q*_*xy*_ (∼0 Å^–1^) is the experimentally inaccessible area.

## Supporting Information Available

Figure S1: AFM-image of a polycrystalline Pt film; Figure S2: individual images for the multimodal microscopy of p6P in Figure 2; Figure S3: optical reflection and polarization microscopy and AFM image for a 20 nm thick p6P film; Figure S4: AFM images and cross sections for various deposition temperatures; Figures S5 and S6: thickness and deposition temperature dependent AFM images; Figure S7: reciprocal space maps of a 20 and 40 nm thick p6P film on Pt; Figures S8 and S9: Raman maps for the various bands for H- and V-polarized light, respectively; and Figure S10: intensity ratio maps for various Raman bands (PDF)

## Abbreviations

UV: ultraviolet
AFM: atomic force microscopy
p6P: *para*-hexaphenylene
OMBD: organic molecular beam deposition
GIXD: grazing incidence X-ray diffraction
XRR: X-ray reflectivity
