# Supplementary material for: Multimodal Microscopy of Partially Oriented para-Hexaphenylene Nanoaggregates
Source: Langmuir. 2024 Nov 23;40(49):25862–70. doi: 10.1021/acs.langmuir.4c03073 (PMC11636257; doi:10.1021/acs.langmuir.4c03073)
Supplement: Supplementary file 1 — la4c03073_si_001.pdf [file la4c03073_si_001.pdf]

Supporting Information: Multimodal Microscopy of Partially Oriented *para*-Hexaphenylene Nanoaggregates

Frank Balzer,[*,†,‖] Mario Fratschko,[‡] Roland Resel,[‡] and Manuela Schiek[*,¶,§]

†*SDU Centre for Photonic Engineering, University of Southern Denmark, Alsion 2, 6400 Sønderborg, Denmark*

‡*Institute of Solid State Physics, Graz University of Technology, Petersgasse 16, 8010 Graz, Austria*

¶*Center for Surface- and Nanoanalytics (ZONA), Johannes Kepler University Linz, Altenberger Str. 69, 4040 Linz, Austria*

§*Institute for Physical Chemistry (IPC) & Linz Institute for Organic Solar Cells (LIOS), Johannes Kepler University, Altenberger Str. 69, 4040 Linz, Austria*

‖*Current address: University of Duisburg-Essen, Faculty of Engineering, Bismarckstr. 81, 47057 Duisburg, Germany*

E-mail: frank.balzer@uni-due.de; manuela.schiek@jku.at

Thin-Film Substrate

The Pt film consists of small grains, as demonstrated by the atomic force microscope (AFM) image in Figure S1(**a**). This has been taken at the part of the sample which was covered by a shadow mask during p6P deposition. In regions in between the p6P aggregates, Figure S1(**b**), the morphology is very similar to that. Therefore it is concluded, that these aggregates also mostly resemble Pt clusters.

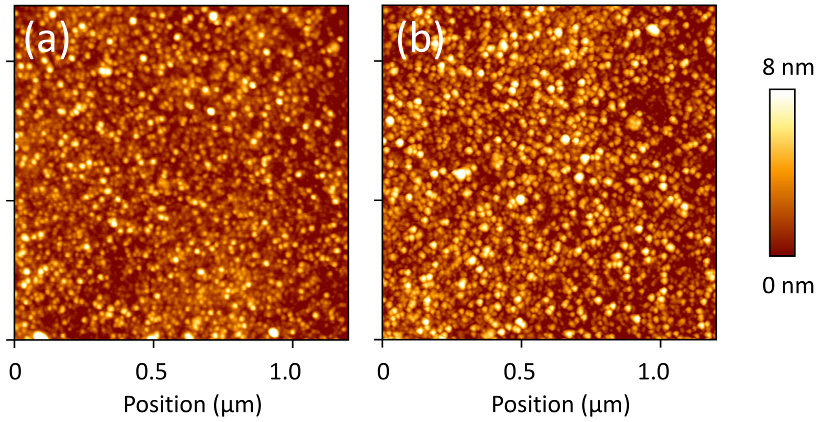

Figure S1: AFM image of the poly-crystalline Pt film (**a**) under the shadow mask and (**b**) in between p6P aggregates.

Morphology of p6P on Pt

Deposition of *para*-hexaphenylene (p6P) on Pt leads to differently shaped aggregates, visible in the AFM images, Figure S3(**a**) and Figure 2, and in the left column of Figure S6. Note that different aggregates lead to vastly different changes in reflectivity, compared to the bare Pt surface. For most of them, reflectivity increases with aggregate height, but also the only a few ten nanometer thick ribbons, type **2** aggregates, show a high reflectivity.

An increase in substrate temperature during deposition leads to less but taller aggregates. This behaviour is demonstrated in Figures S4 and S5. In Figure S4, AFM images for 7 nm thick p6P films deposited at 180 °C, (**b**) 200 °C, and (**c**) 220 °C are presented together with cross-sections along a horizontal line. For the same samples on a larger scale, optical images

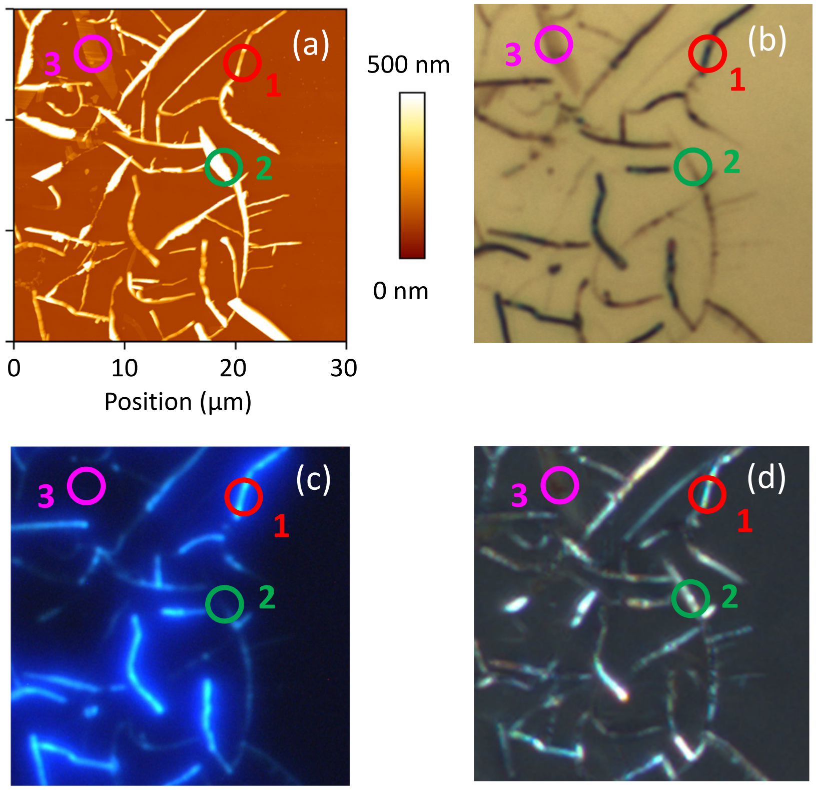

Figure S2: Multi-modal microscopy of p6P aggregates. (**a**) AFM-image of nominally 20 nm p6P on Pt, deposited at 200 °C. The corresponding optical reflection microscope image, the fluorescence microscope image, and a white light microscope image with crossed polarizers are shown in (**b**), (**c**), and (**d**), respectively. Different aggregate types are marked by numbers **1**, **2**, and **3**. The optical images have been overlayed on the AFM image in Figure 2.

without polarizers and with two crossed polarizers are presented in Figure S5. Such a temperature dependence is typical for organic fiber growth and has been documented in the literature various times before.[1–4]

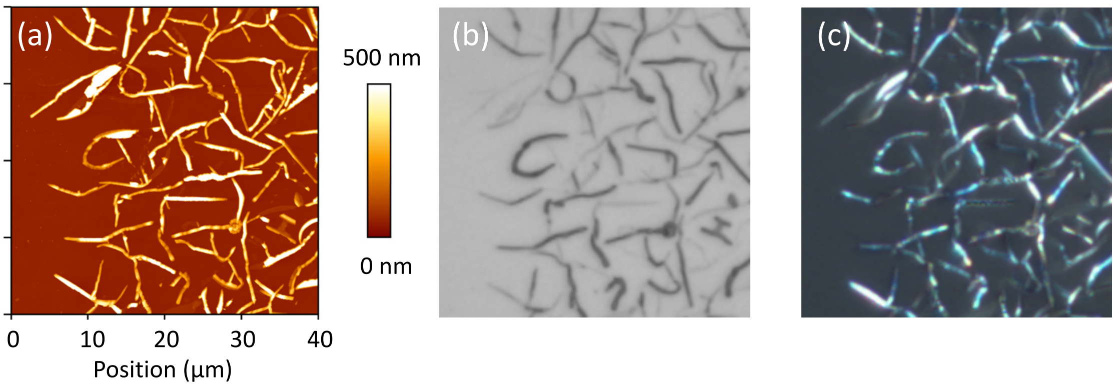

Figure S3: AFM image (**a**) and corresponding white light reflection microscope images (**b,c**) for a 20 nm thick p6P film on Pt. In (**b**), unpolarized light is used, in (**c**) the sample is placed in between two crossed polarizers. The left part of the sample was covered by a shadow mask during p6P deposition.

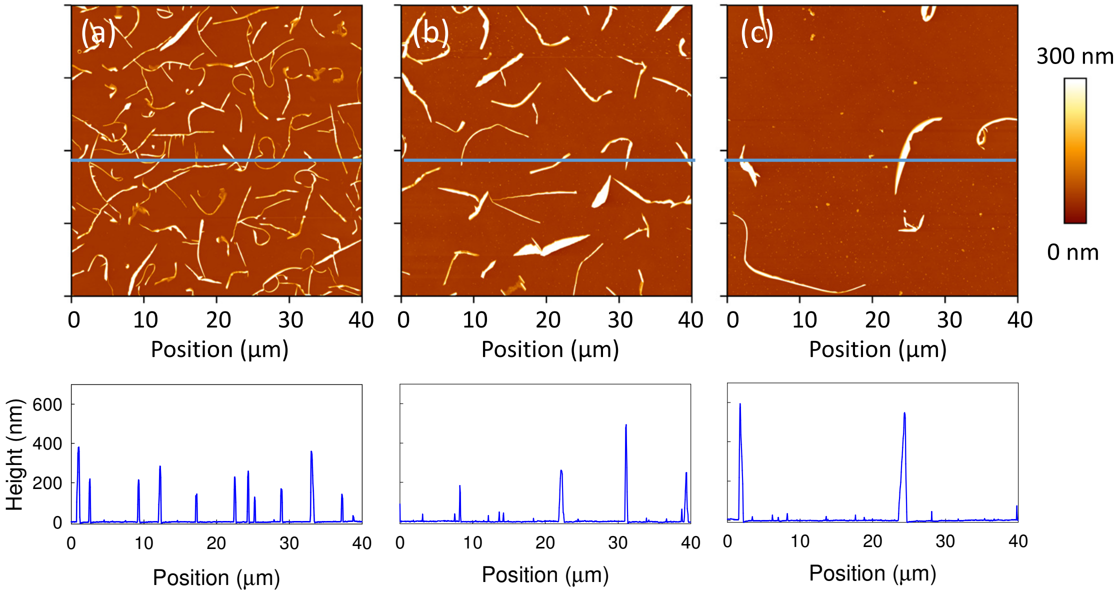

Figure S4: AFM images for 7 nm thick p6P films on Pt, deposited at (**a**) 180 °C, (**b**) 200 °C, and (**c**) 220 °C. The cross sections along the blue lines demonstrate the increase in height with increasing deposition temperature, and the decrease of aggregate number density.

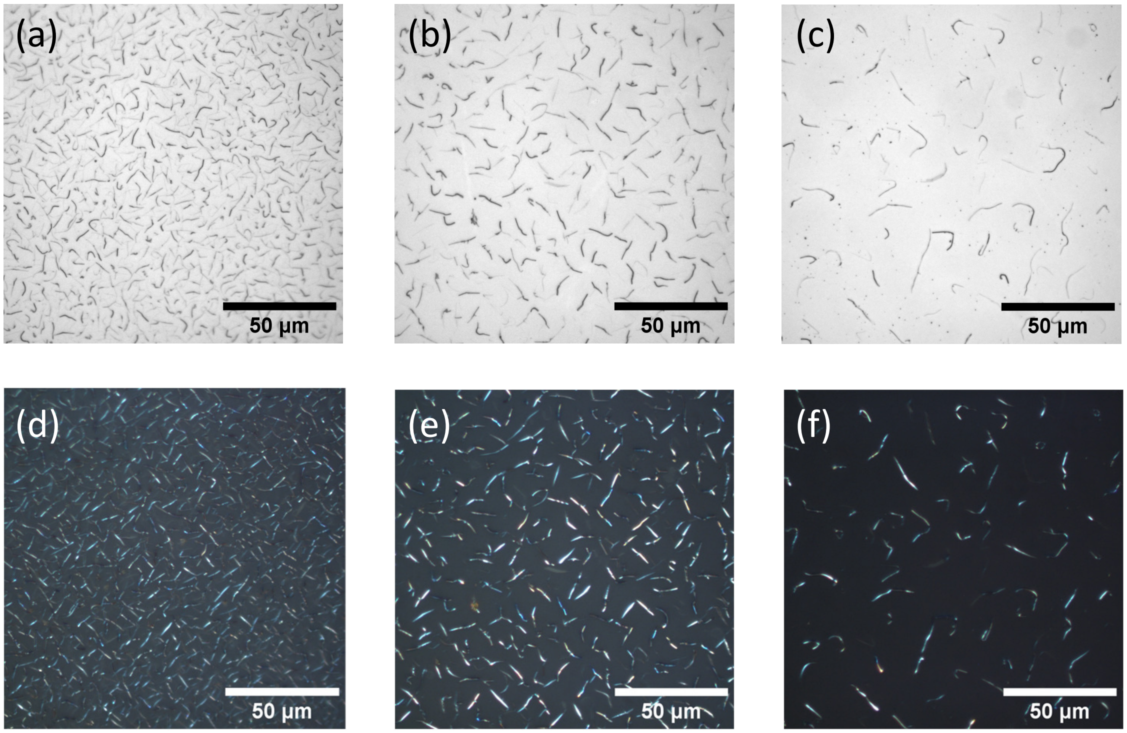

Figure S5: Gray-scale optical reflection microscope images using either no polarizer (upper row) or two crossed polarizers (lower row) for 7 nm thick p6P films on Pt, deposited at (**a,d**) 180 °C, (**b,e**) 200 °C, and (**c,f**) 220 °C.

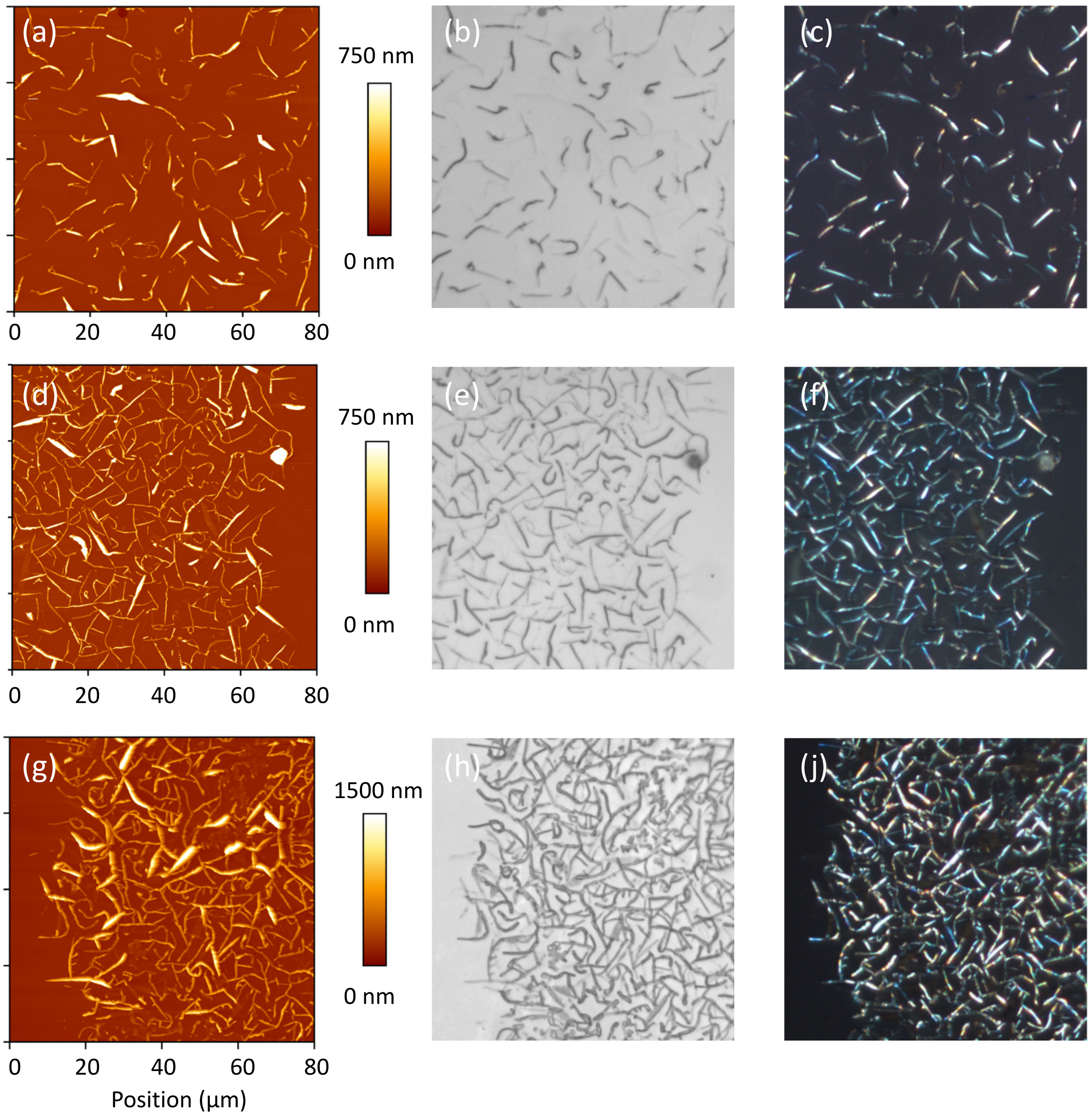

Figure S6: AFM images (left column) and corresponding gray-scale optical reflection microscope images (middle and right column) for a 7 nm thick p6P film on Pt (upper row), a 20 nm thick p6P film (middle row), and a 40 nm thick p6P film (lower row). In the middle column, white unpolarized light is used, in the right column white light with two crossed polarizers. For the two thick films, part of the sample was covered by a shadow mask during p6P deposition. In all cases, the sample temperature during deposition was 200 °C.

Grazing Incidence X-Ray Diffraction (GIXD)

The experimental diffraction pattern are depicted in reciprocal space. The result is presented as function of the in-plane part of the scattering vector (q_{xy}) and of the out-of-plane part of the scattering vector (q_z). The results at negative and positive values of q_{xy} give identical diffraction information. For clarity, the peak assignments are given only at one side. The observed diffraction peaks are drawn by quadrants, indicating the peak positions. The strong diffraction peaks at $q_{xy}/q_z = \pm 1.63\,\text{Å}^{-1}/1.17\,\text{Å}^{-1}$, arise from equivalent 111 Bragg peaks of the silicon substrate.

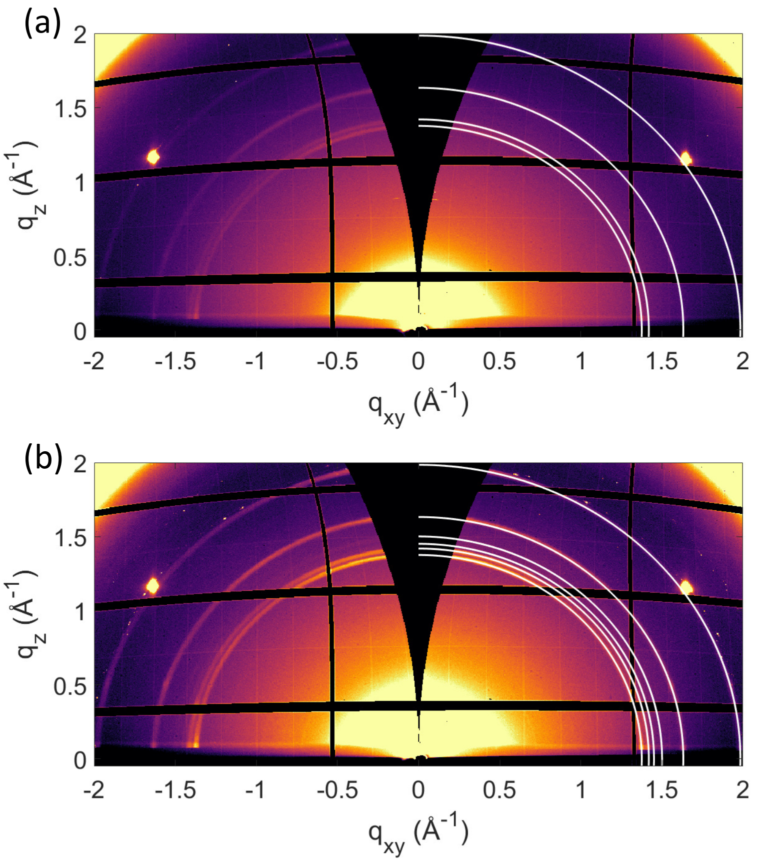

Figure S7: (**a**) Reciprocal space map of a 20 nm thick p6P film on Pt (incident angle 0.8°). The Debye-Scherrer rings are characteristic for the p6P herringbone structure. $(1\,1\,\bar{1})$: lattice spacing $d = 4.59\,\text{Å}$, length of the scattering vector $q = 2\pi/d = 1.37\,\text{Å}^{-1}$; $(2\,1\,\bar{1})$: $d = 4.46\,\text{Å}$, $q = 1.41\,\text{Å}^{-1}$; $(3\,0\,\bar{2})$: $d = 3.88\,\text{Å}$, $q = 1.62\,\text{Å}^{-1}$; $(3\,1\,\bar{2})$: $d = 3.16\,\text{Å}$, $q = 1.99\,\text{Å}^{-1}$. (**b**) Same as (**a**), but for a 40 nm thick p6P film. Additional rings correspond to $(6\,0\,0)$: $d = 4.33\,\text{Å}$, $q = 1.45\,\text{Å}^{-1}$ and $(3\,1\,\bar{1})$: $d = 4.22\,\text{Å}$, $q = 1.49\,\text{Å}^{-1}$. White circles highlight the expected positions of the reflections from the herringbone p6P structure.

Raman Maps

Raman maps for the various bands and for two different polarization directions of the Raman laser ($\lambda_{exc} = 532\,\text{nm}$) are found in Figure S8 for H-polarized light and in Figure S9 for V-polarized light. A red and green circle mark type **1** and type **2** aggregates, respectively. The maps demonstrate the varying sensitivity to the aggregate types **1** and **2**, and the sensitivity to excitation polarization with respect to the aggregate direction. Bands A, B, and C are most intense for the excitation polarization perpendicular to the type **1** aggregate direction. Bands D and E show a lower aggregate sensitivity, but have still a maximum if they excitation polarization is perpendicular to the long fiber axis of type **1** aggregates.

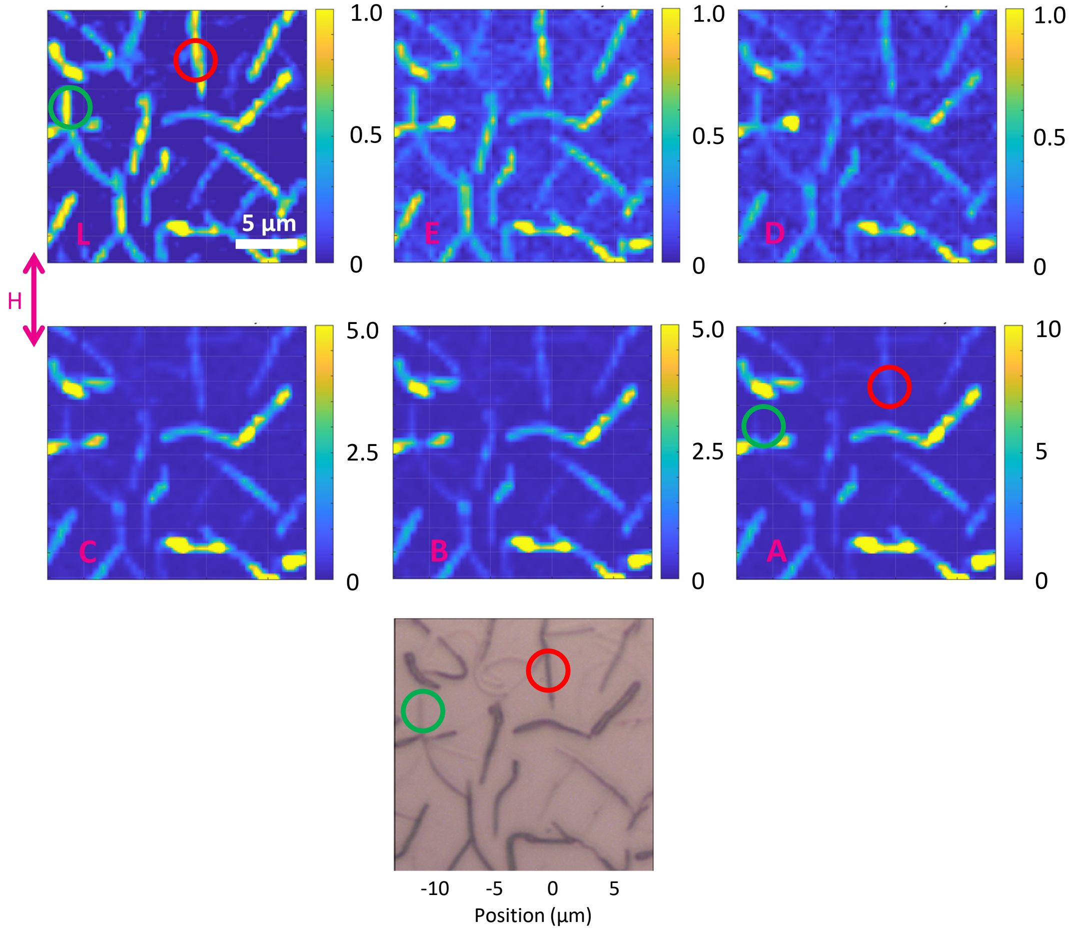

Figure S8: Raman maps of the librational band (L) and for the bands A-E. The excitation light was H-polarized. The circles mark specific aggregates, see text. An optical reflection microscope image of the same area is also shown.

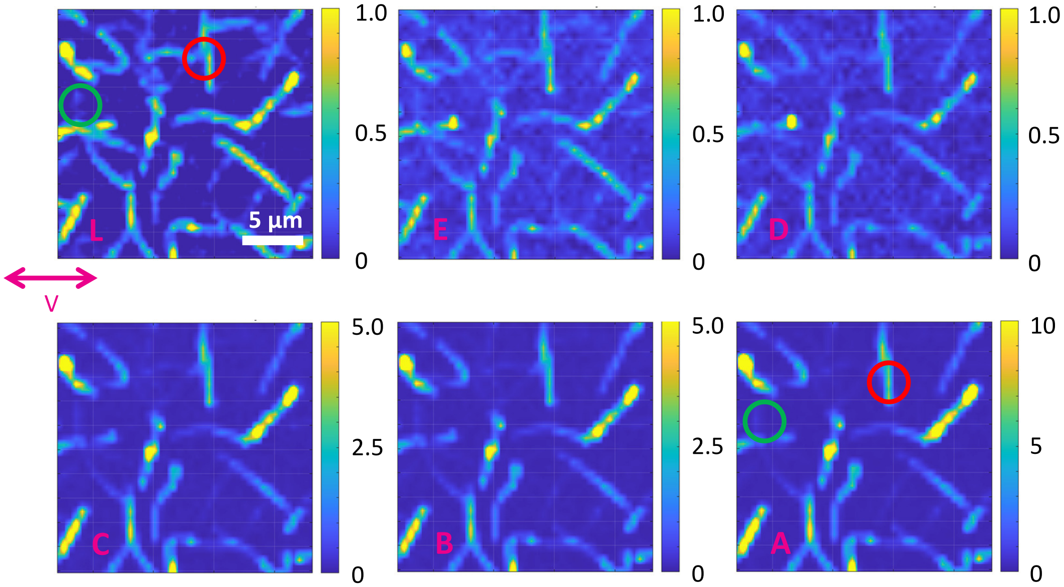

Figure S9: The same as Figure S8, but for V-polarized excitation light.

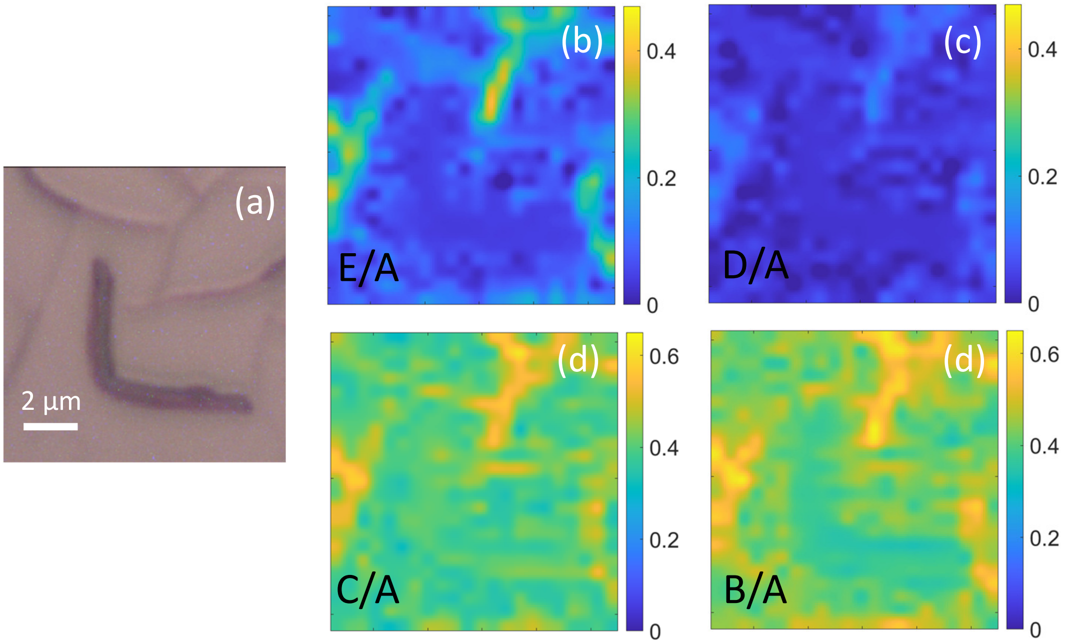

Figure S10: Ratios of the integrated band intensities (sums of H- and V-polarization) of the E-, D-, C-, and B-band relative to the intensity of the A-band (a) for the sample from Figure 7. The corresponding optical reflection microscopy image is shown in (a). The intensity ratios are larger for the type **2** aggregates than for the type **1** aggregate, i.e. for the dark L-shaped fiber,

The ratios between the different band intensities in Figure S10 demonstrate that even so the positions of the Raman lines are identical for the different aggregate types **1** and **2**, the relative intensities of the various Raman bands differ, hinting to different molecule orientations.

References

(1) Kankate, L.; Balzer, F.; Niehus, H.; Rubahn, H.-G. From Clusters to Fibers: Parameters for Discontinuous *p*-6P Thin Film Growth. *J. Chem. Phys.* **2008**, *128*, 084709.

(2) Rubahn, H.-G., Sitter, H., Horowitz, G., Al-Shamery, K., Eds. *Interface Controlled Organic Thin Films*; Springer Proceedings in Physics; Springer: Berlin, 2009; Vol. 129; ISBN: 978-3-540-95929-8.

(3) Simbrunner, C.; Nabok, D.; Hernandez-Sosa, G.; Oehzelt, M.; Djuric, T.; Resel, R.; Romaner, L.; Puschnig, P.; Ambrosch-Draxl, C.; Salzmann, I.; Schwabegger, G.; Watzinger, I.; Sitter, H. Epitaxy of Rodlike Organic Molecules on Sheet Silicates – A Growth Model Based on Experiments and Simulations. *J. Am. Chem. Soc.* **2011**, *133*, 3056 – 3062.

(4) Sitter, H., Draxl, C., Ramsey, M., Eds. *Small Organic Molecules on Surfaces*; Springer Series in Materials Science; Springer: Berlin, 2013; Vol. 173.
